# Microfabrication of Microlens by Timed-Development-and-Thermal-Reflow (TDTR) Process for Projection Lithography

**DOI:** 10.3390/mi11030277

**Published:** 2020-03-07

**Authors:** Jun Ying Tan, Gyuhyeong Goh, Jungkwun Kim

**Affiliations:** 1 Department of Electrical and Computer Engineering, Kansas State University, Manhattan, KS 66506, USA; justintan@ksu.edu; 2 Department of Statistics, Kansas State University, Manhattan, KS 66506, USA; ggoh@ksu.edu

**Keywords:** microlens, microlens array, timed-development, thermal reflow, lithography, photoreduction

## Abstract

This paper presents a microlens fabrication process using the timed-development-and-thermal-reflow process, which can fabricate various types of aperture geometry with a parabolic profile on a single substrate in the same batch of the process. By controlling the development time of the uncrosslinked negative photoresist, a state of partial development of the photoresist is achieved, called the timed development process. The thermal reflow process is followed after the timed development, which allows the photoresist to regain its liquid state to form a smooth meniscus trench surrounded by a crosslinked photoresist sidewall. Microlens with larger aperture size forms deeper trench with constant development time. With constant aperture size, longer developing time shows deeper meniscus trench. The depth of the meniscus trench is modeled in the relationship of the development time and aperture size. Other characteristics for the microlens including the radius of curvature, focal length, and the parabolic surface profile are modeled in the relationship of the microlens thickness and diameter. Microlens with circular, square, and hexagonal bases have been successfully fabricated and demonstrated where each geometry of the lens-bases shows different fill factors of the lens arrays. To test the fabricated lenses, a miniaturized projection lithography scheme was proposed. A centimeter-scale photomask pattern was photo-reduced using the fabricated microlens array with a ratio of 133, where the smallest linewidth was measured as 2.6 µm.

## 1. Introduction

Microlens arrays (MLAs) has been widely used in various optoelectronics technologies as it is very versatile and relatively simple at manipulating light. Recent reports have suggested that high surface fill factor MLAs as the key component in several developing modern technologies. Examples such as imaging systems in light-field cameras, 3D light field cameras, and compound eye camera [1,2,3], sensing devices such as charge-coupled devices (CCD) and atmospheric wavefront sensors [3], and a light source device such as organic light-emitting diodes (OLEDs) [3]. Other notable examples that require single microlens or sparsely filled MLA are waveguides, optical interconnect systems and optical fibers for optimization of optical coupling and endoscope for biomedical instruments [3,4]. These devices are highly demanded nowadays and expected to be better by having smaller and slimmer designs to achieve better comforts and safety, for example, wearable, swallow-able, pocket-size and so on. However, to satisfy all these demands, a low cost, repeatable, and scalable fabrication process that can fabricate both high and low surface fill factor MLA still remains challenging. 

Recent reports have demonstrated various fabrication processes for MLAs including ink-jetting [5], excimer laser ablation [6,7], excimer laser micromachining [8], grayscale lithography [9], and photoresist thermal reflow [10]. However, most of them still face common problems such as numerous, complex fabrication steps, which results in high cost and time-consuming. Timed-development-and-thermal-reflow (TDTR) process was reported to fabricate curved structures using simple microlithography steps [11,12,13] and also presented the modeling equations to support the experimental results [14]. The versatility of the TDTR process showed the ease of fabrication by following the conventional lithography process and demonstrated its great potential for curved trenches in microfluidic channels built-in real 3D shapes and both plano-convex and concave microlenses with different sizes. 

Building on top of the previous reports [13,14], this paper further characterized and modeled the microlens with updated modeling equations for predicting the characteristics of the microlens with elliptical paraboloid profile, which have been suggested by many studies that such microlens profile can greatly suppress the spherical aberration and improve the imaging quality [15,16]. This paper demonstrated a novel fabrication of microlenses with different base geometries and sizes on the same substrate with single UV exposure, which represents the uniqueness of the TDTR process. The usefulness of the TDTR process demonstrated the fabricated MLAs with high uniformity and high surface fill factor, which has been one of the common challenges with other microlens fabrication process. A projection lithography scheme was demonstrated as a practical application of the microlens array for projecting an array of photo-reduced photopatterns, showing great potential in microstructure fabrication.

## 2. Timed-Development-and-Thermal-Reflow (TDTR) Process 

**Figure 1:**
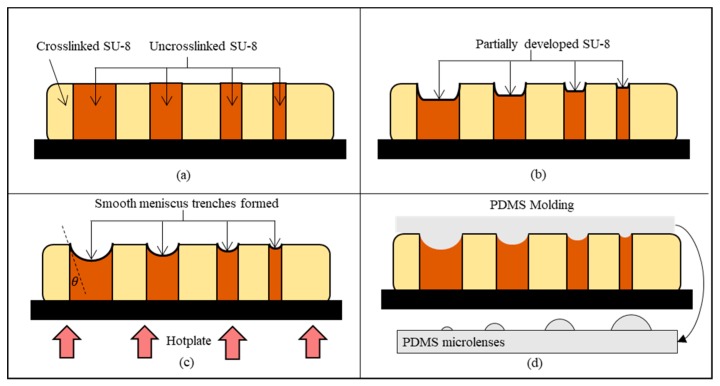
Timed-development-and-thermal-reflow (TDTR) process: (**a**) SU-8 with crosslinked and uncrosslinked region after UV exposure; (**b**) timed development process to partially develop photoresist; (**c**) thermal reflow process to create smooth meniscus trench; (**d**) Polydimethylsiloxane (PDMS) molding to create microlenses.

A curvature fabricated by the TDTR process is suitable for a microlens application. The surface profile of the curvature can be predicted based on the reduced amount of uncrosslinked photoresist in the development process and the curvature reformation in the post thermal reflow process as described in Figure 1. An area of the solvent-dissolvable SU-8 photoresist was defined by the unexposed SU-8 area from the UV lights while the UV exposed area becomes non-dissolvable serving as the sidewall structures as shown in Figure 1a. Figure 1b describes the partial development process, which controls an amount of etched-out SU-8 by the developing time. The trenches are formed in this process with a flat bottom and steep inclination angle against the sidewall. The remaining uncrosslinked SU-8 is reformed into a liquid state from the thermal reflow process for smooth curvatures as shown in Figure 1c. Figure 1d shows the micro-molding process using Polydimethylsiloxane (PDMS) to fabricate the microlenses. 

This relationship was also previously discussed in terms of the contact angle variation between the crosslinked photoresist and the reflowed uncrosslinked surface [14] using the Young-Laplace equation [17] as shown below:

(1) $${\left(\frac{t_{c}}{a}\right)}^{2}=1-\sin\left(\theta \right),$$

where $t_{c}$ is the depth of meniscus trench, θ is the contact angle, and a is the capillary constant, which can be calculated from the radius of curvature of the meniscus and the capillary length [18].

Since the diffusion of the uncrosslinked SU-8 determines the amount of the etched-out SU-8 during the developing process, the more time and the larger contact area results in more amount of the etched-out SU-8. These unique characteristics can produce various geometries of the trench and thereby various microlenses after micro-molding. Equation (1) predicts the surface profile of the trench in terms of the developing time and the contact angle. Since Equation (1) does not directly explain the surface profile of the trench as a geometry of an optical lens, further investigation on Equation (1) is necessary to make it suitable for an optical lens application.

## 3. Modeling

**Figure 2:**
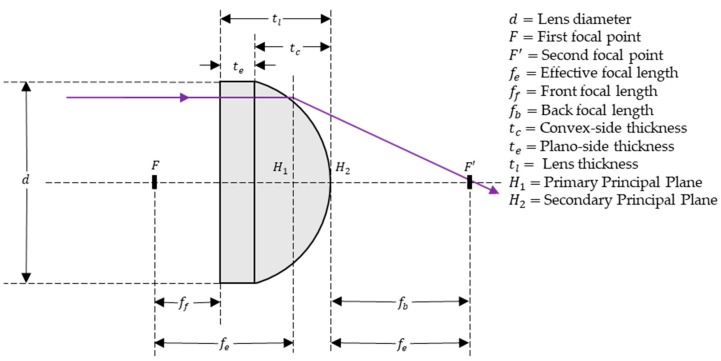
Characteristics of the plano-convex microlens.

In the fabricated trench by the TDTR process, the depth of meniscus trench and lens thickness can be interpreted as the same parameter $t_{c}$. The depth of the meniscus trench is used when discussing the microlens mold, while lens thickness is used when discussing the microlenses. Aperture size and lens diameter are the same parameter d and have the same value where the aperture size is used when discussing the microlens mold and lens diameter is used when discussing the microlenses. The optical parameters of the microlens are described in Figure 2. 

### 3.1. Depth of the Meniscus Trench

**Figure 3:**
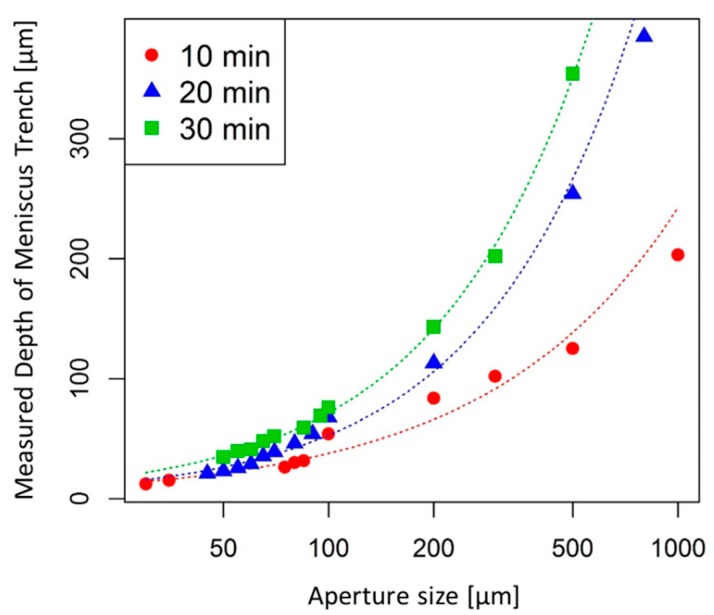
Relationship between the measured depth of meniscus trench for various aperture diameter at three different development time (10, 20, and 30 min).

The depth of the meniscus trench ($t_{c}$) can be determined by the development time and the aperture size. Figure 3 shows the measured results of the depth of the meniscus trench with various aperture sizes for three different development times (10, 20, and 30 min). This plot was generated based on statistical data obtained from the experiments, and the R^2^ value was 0.9446, 0.9871, and 0.9820 for 10, 20, and 30 min development time respectively, which provides a good graphical approximation on the depth of meniscus trench based on aperture sizes and development time.

To provide a mathematical method to better predict the depth of the meniscus trench, a nonlinear regression analysis method [19] was adopted to model the depth of meniscus trench based on experimentally measured data. This analysis described the relationship between the depth of meniscus trench in µm and developing time in minute with varying aperture size in µm using the equation shown below:

(2) $$\log\left(t_{c}\right)=b_{1}\log\left\{\log\left(d\right)\right\}+b_{2}\log\left(t\right)+b_{3}\log\left\{\log\left(d\right)\right\}\log\left(t\right).$$

**Figure 4:**
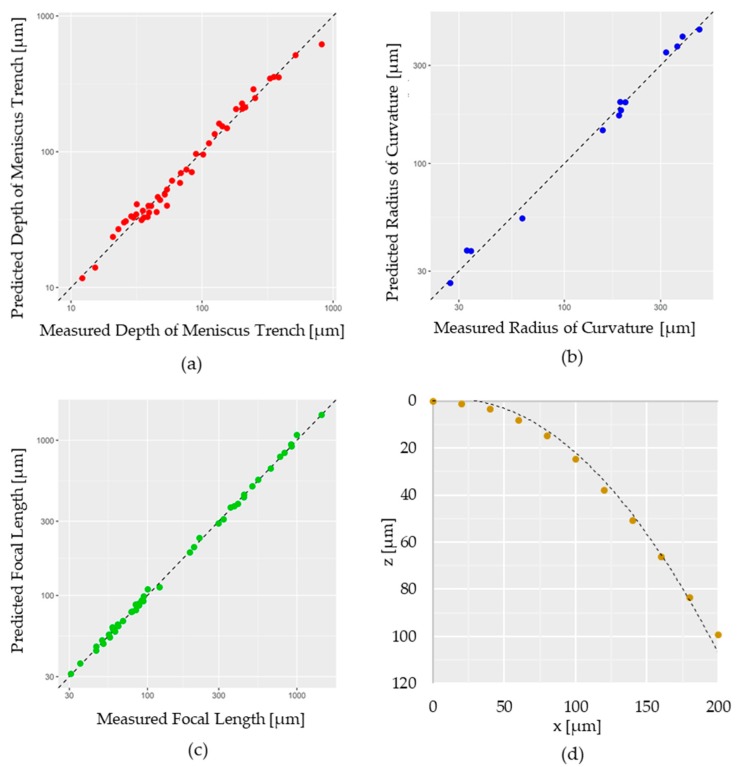
Scatter plots comparing the measured data against the predicted data using the modeling equations. The color dots represent the measured data, and the dashed line represents the predicted data: (**a**) the depth of meniscus trench; (**b**) the radius of curvature; (**c**) focal length; (**d**) surface profile.

Thereby, the depth of the meniscus trench can be defined as:

(3) $$t_{c}={\left\{\log\left(d\right)\right\}}^{b_{1}+b_{3}\log\left(t\right)}t^{b_{2}},$$

where $b_{1}$ = 1.574077, $b_{2}$ = −1.089105, $b_{3}$ = 1.078329, and log denotes the natural logarithm (ln). The coefficients $b_{1}$, $b_{2}$ and $b_{3}$ in Equation (3) were obtained by minimizing the least square error. The least-square estimates are computed by ln function in R, a software environment for statistical computing and graphics:

(4) $$R\left(b_{1},b_{2},b_{3}\right)=\sum_{i=1}^{n}{\left[\log\left(t_{c,i}\right)-b_{1}\log\left\{\log\left(d_{i}\right)\right\}+b_{2}\log\left(t_{i}\right)+b_{3}\log\left\{\log\left(d_{i}\right)\right\}\log\left(t_{i}\right)\right]}^{2},$$

where $t_{c,i}$, $d_{i}$ and $t_{i}$ indicate the observed values of $t_{c}$, d, and t at the *i*-th experiment, and n is the total number of observations. Figure 4a demonstrates that the predicted depth of the meniscus trench from Equation (3) well explained the variability of the measured depth of the meniscus trench.

### 3.2. Effective Focal Length

The effective focal length of the paraboloid plano-convex microlens can be defined by the lens maker’s equation:

(5) $$\frac{1}{f_{e}}=\left(n-1\right)\left(\frac{1}{R_{1}}-\frac{1}{ROC}\right),$$

where $f_{e}$ is the effective focal length, *n* is the refractive index of PDMS, $R_{1}$ is the radius of curvature of the plane surface of the microlens, which can be assumed as infinity, and ROC is the radius of curvature at the vertex of the parabolic surface of the microlens. To obtain the radius of curvature of the parabolic surface, a linear regression analysis was adopted, and the radius of curvature can be described as:

(6) $$\log\left(ROC\right)=a_{0}+a_{1}\log\left(d\right)+a_{2}\log\left(t_{c}\right),$$

where ROC is the radius of curvature, $t_{c}$ is the thickness of the microlens, d is the microlens diameter, and log denotes the natural logarithm (ln), note that these parameters are labeled in Figure 2. Thereby, the curvature radius of the microlens can be modeled as:

(7) $$ROC=e^{a_{0}}{\left(d\right)}^{a_{1}}{\left(t_{c}\right)}^{a_{2}},$$

where $a_{0}=-1.17852$, $a_{1}=1.31930$, $a_{2}=-0.27266$, e is the exponential constant, d is the microlens diameter, and $t_{c}$ is the microlens thickness. Since PDMS (Sylgard 184) was used as the lens material in the fabrication process for MLAs with a constant refractive index of 1.4483 in 405 nm (“H-line”) [20], the effective focal length can be derived from Equations (5) and (7) as:

(8) $$f_{e}=\frac{ROC}{n-1}=\frac{e^{a_{0}}{\left(d\right)}^{a_{1}}{\left(t_{c}\right)}^{a_{2}}}{n-1}.$$

Figure 4b,c demonstrate the predicted radius of curvature and focal length are well-matched with the measured radius of curvature and focal length, indicating that the model equation accurately explained the focal length with the variable of lens thickness and lens diameter.

### 3.3. Surface Profile

The surface profile (z) of the microlenses can be obtained from the elliptical paraboloid equation shown below:

(9) $$z=t_{c}-c_{0}\frac{t_{c}{}^{c_{1}}}{d^{c_{2}}}x^{c_{3}}-c_{0}\frac{t_{c}{}^{c_{1}}}{d^{c_{2}}}y^{c_{3}},$$

where x, y and z are the coordinates on the parabolic surface of the microlens, $t_{c}$ is the microlens thickness, and d is the microlens diameter. By setting $t_{c}$ and d as constant and z as a function of x and y, $c_{0}$, $c_{1}$, $c_{2}$, and $c_{3}$ can be estimated using excel solver by minimizing the sum of squared error, which $c_{0}$ = 3.79583504, $c_{1}$ = 0.742831171, $c_{2}$ = 2.153050191, and $c_{3}$ = 2.407866931 were obtained. Figure 4d demonstrates the predicted surface coordinates of the microlens well-matched with the measured surface coordinates based on Equation (9), which implies that the model equation accurately explained the surface profile with variable microlens thickness and diameter.

### 3.4. Modeling Equation Summary

The subsection summarizes the estimated coefficient and constants for the modeling equations. Table 1 shows the summary of nonlinear regression analysis for deriving the relationship between the depth of meniscus trench and development time with various aperture size as described in Equation (3). All the estimated coefficients are statistically significant at the significance level of α = 0.0001. The strength of the relationship between the observed data and the fitted regression model was measured by computing the coefficient of multiple determination, often referred to as R^2^. The computed R^2^ was 0.9993, which implies that 99.93% of the variation in the depth of the meniscus trench can be explained by the fitted model in Equation (3).

Table 2 shows the summary of linear regression analysis for deriving the relationship between the radius of curvature of the microlens at various microlens thickness and diameter as described in Equation (5). The computed R^2^ was 0.9935, which implies that 99.35% of the variation in the radius of curvature can be explained by the fitted model in Equation (5). As the focal length is simply a function of the radius of curvature, Table 2 also indirectly implies the accuracy of Equation (7) at predicting the focal length. 

Table 3 shows the estimated result of the constant in Equation (8) using excel solver by minimizing the sum of squared error. The computed R^2^ was 0.9987, which implies that 99.87% of the variation in the surface profile can be explained by the fitted model in Equation (8). 

## 4. Fabrication Process

**Figure 5:**
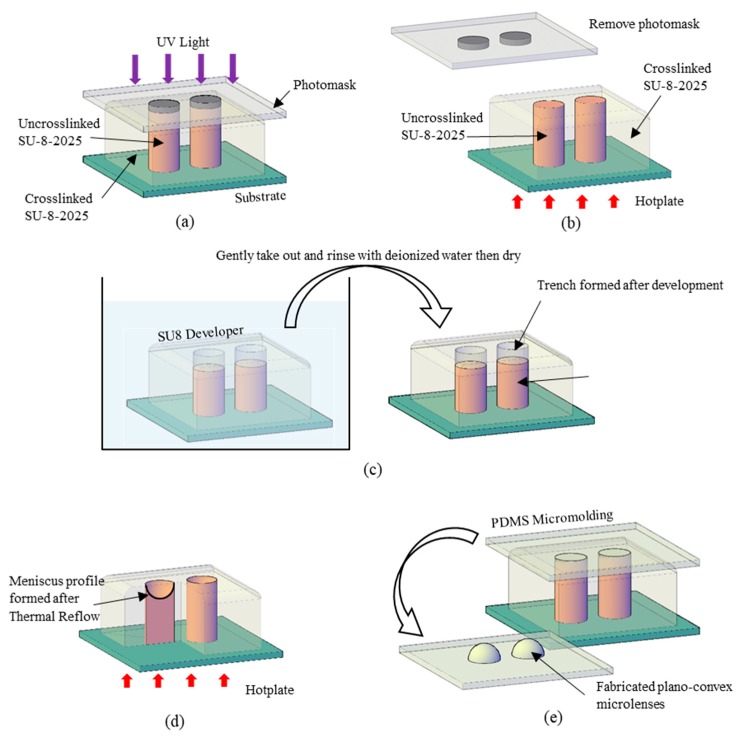
Major fabrication steps of the TDTR process: (**a**) UV exposure with a pre-designed photomask placed on the photoresist (SU-8); (**b**) remove mask after UV exposure then post-exposure-bake; (**c**) Timed-Development in SU-8 developer (PGMEA); (**d**) Thermal-Reflow to form meniscus profile; (**e**) PDMS micro-molding for microlens arrays.

An example of TDTR fabrication process is described as shown in Figure 5. A glass slide was prepared as the substrate. The substrate was cleaned with organic solvents (acetone, methanol, and isopropanol) and baked to remove surface moisture at 95 °C for 10 min using a hotplate. A negative photoresist, SU-8 2025, was used in this fabrication process because of its excellent imaging and fast drying characteristics. 1.14 grams of photoresist was poured on a 1 × 1 in^2^ substrate and soft-baked at 65 °C for 20 min and increased to 95 °C for 12 h with a temperature ramping speed of 200 °C/h. After the soft-baking process, the sample was cooled down to room temperature with a temperature ramping speed of 200 °C/h. The photoresist shrunk slightly after cool down as most of the solvent gradually evaporated from the photoresist during the soft bake process, forming a 1 mm thick photoresist on the substrate. Note that the amount of photoresist was verified based on a weighing and dispense method. Also, note that in this experiment, 1 mm would be set as the maximum lens thickness possibly fabricated. A photomask with an array of various holes was placed on top of the photoresist, and the UV exposure process was followed where the exposure dose of 2.4 J/cm^2^ (LS 30, OAI Inc., Chicago, IL, USA) was applied as depicted in Figure 5a. The photomask was removed after the exposure process, and the sample was transferred to a hotplate for post-exposure-bake (PEB) at 65 °C for 20 min and increased to 95 °C for an hour with a temperature with ramping speed of 200 °C/h to crosslink all the exposed photoresist as described in Figure 5b. The sample was gradually cooled down to room temperature to prevent thermal-mismatch cracks on the photoresist. The sample was then developed by dipping it into SU8 developer (propylene glycol monomethyl ether acetate solution, PGMEA) for partial development depicted in Figure 5c. This partial development was time-controlled to achieve the desired amount of photoresist development. Note that this step is crucial as it determines the shape of the MLAs. The sample was gently taken out from the developer and then transferred to a hotplate for thermal reflow at 95 °C (200 °C/h temperature ramping speed) for an hour to form a smooth meniscus profile as depicted in Figure 5d. After completely cooling down the sample, UV flood exposure was followed and baked at 95 °C for an hour to finalize the meniscus profile. After the substrate has cooled down to room temperature, the mold for plano-convex MLAs was completed. Polydimethylsiloxane (PDMS) was used as the material to fabricate MLAs, considering that its micro-to-nano-molding characteristics and its high optical transparency to UV light and visible light. A micro-molding process was conducted using PDMS; 10 parts of the PDMS base were well mixed with 1 part of the curing agent then degassed in a vacuum chamber for 10 min. A clear, bubble-free PDMS solution was poured on the mold then baked in an oven at 80 °C for 3 h. After the PDMS was completely cured, it was carefully peeled off to complete the MLAs, as depicted in Figure 5e.

## 5. Results

**Figure 6:**
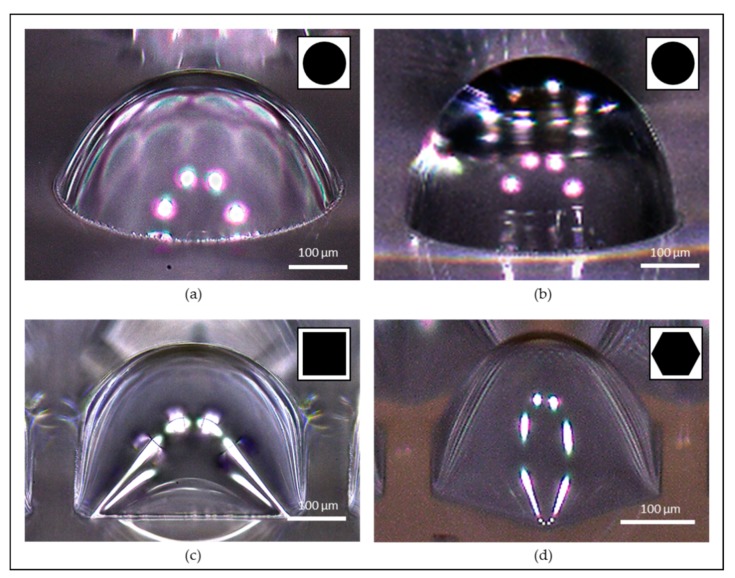
Microlens arrays (MLAs) fabricated using the TDTR process with varying development time or aperture geometry. Circular aperture microlens with a diameter of 500 µm with: (**a**) 10 min development; (**b**) 20 min development; (**c**) square aperture and the side length of 500 µm with 10 min development; (**d**) hexagonal aperture with an inner circle diameter of 300 µm with 20 min development.

Four batches of MLAs were successfully fabricated with varying development time or photomask geometry (circular, square and hexagonal) as shown in Figure 6, where the close-up view of the fabricated microlens is demonstrated with the mask pattern (inset) that was used in the fabrication. Note that the white dots in Figure 6 are the reflection of the LED from the optical microscope that was used to capture the images.

### 5.1. Varying Development Time

Figure 6a,b show two different results of microlenses by differentiating the developing time of 10 min and 20 min with the same photomask pattern. The microlens in Figure 6a has a diameter of 500 µm and the lens thickness of 143.18 µm, while Figure 6b shows a diameter of 500 µm and the lens thickness of 253.97 µm. The fabrication result clearly explains that the microlens with longer development time shows larger lens thickness, showing that this fabrication process can easily customized microlens by simply controlling the development time to determine the thickness of the microlens in a given diameter. Furthermore, this result also demonstrated the TDTR process is more versatile than other fabrication processes that strongly rely on the surface tension of the material to create the spherical or parabolic profile of the microlens [21].

### 5.2. Varying Base Geometry

Figure 6c,d show the results of microlenses with square and hexagonal base apertures. Figure 6c shows the 10 min development result of a square base microlens with a side length of 500 µm, and the thickness of the lens was measured to be 166.53 µm. Note that the thickness of the square microlens is slightly thicker than the circular microlens, this is because the contact area of the square base with the developer was slightly larger than the circular base, which caused a slight change in the developing rate and resulted in slightly thicker microlens. Figure 6d shows 20 min development result of a hexagonal aperture with an inner circular diameter of 300 µm, and the thickness of the lens was measured as 178.84 µm. The results show that the TDTR process is capable of fabricating various types of microlens by simply changing the base aperture to satisfy the various need in different types of microlenses for different purposes.

### 5.3. Lens Fill Factor

A lens fill factor on a unit surface has been investigated as it is important when fabricating MLAs as higher surface fill factor can result in more number of microlenses. Typically, the lens fill factor can be defined as:

(10) $$Suface\;Fill\;Factor=\frac{Area\;of\;the\;MLAs}{Area\;of\;the\;whole\;surface}\times 100\%.$$

**Figure 7:**
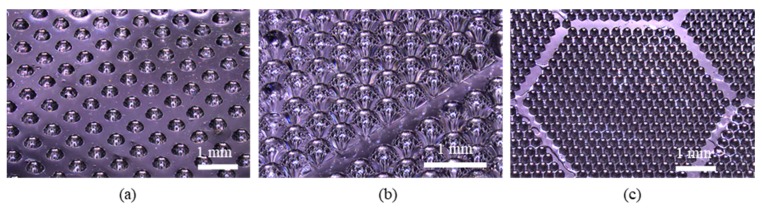
Overview of MLAs with: (**a**) circular aperture; (**b**) square aperture; (**c**) hexagonal aperture.

Based on Equation (10), the surface fill factor with the circular aperture is hard to reach higher than 80% as the voids among the circular apertures are inevitable. To achieve the higher surface fill factor, the different aperture is often suggested, for example, square and hexagonal apertures can be easily filled up the surface. Figure 7 demonstrated the MLAs with different apertures. Figure 7a shows an MLA with a low fill factor of 30.7% with sparsely designed circular aperture whereas Figure 7b,c show MLAs with square and hexagonal the achieving fill factor up to 82.6% by changing the aperture geometry and layout of the MLAs. With this fabrication process, fill factor can be easily modified by changing the mask pattern layout and aperture geometry, this allows vast freedom while designing the MLAs to fit various purposes and accommodate different devices. Low fill factor MLAs are suitable for separating the MLAs into individual microlenses, which can be used on micro-optical fibers or probes. High fill factor MLAs can be used on CCD arrays or digital screen for converging light onto the photoactive region.

### 5.4. Uniformity Test

To discuss the uniformity of the lens thickness of three batches of MLAs with different aperture geometries, all the lens thickness of the fabricated MLAs were measured and used to calculate the mean, variance, and standard deviation. The calculated data was then substituted into the Equation (11) to derive the uniformity of the MLAs.

(11) $$Uniformity\;\%=\left(1-\frac{\sigma }{Mean}\right)\times 100\%,$$

where σ is the standard deviation of the measured lens thickness. 

Table 4 shows that the uniformity of the MLAs fabricated using this fabrication process is up to 97.56%, showing high throughput, reliability, repeatability, and even manufacturability characteristics of this fabrication process. As compared to other studied fabrication processes that have reported 31 µm for the standard deviation of circular microlens thickness [22], the TDTR process shows the better result as 4.96 µm in terms of microlens thickness consistency throughout the whole sample. 

## 6. Application

**Figure 8:**
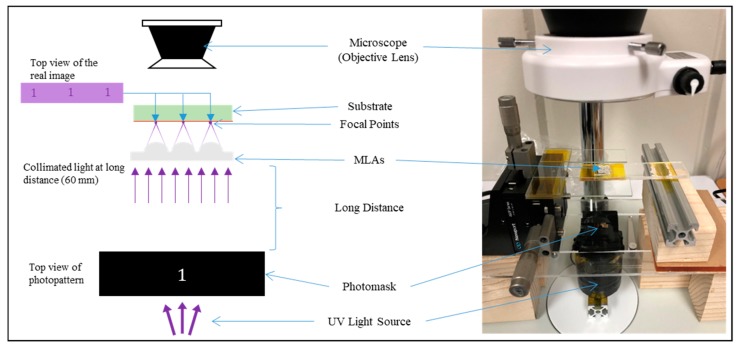
Projection lithography scheme.

**Figure 9:**
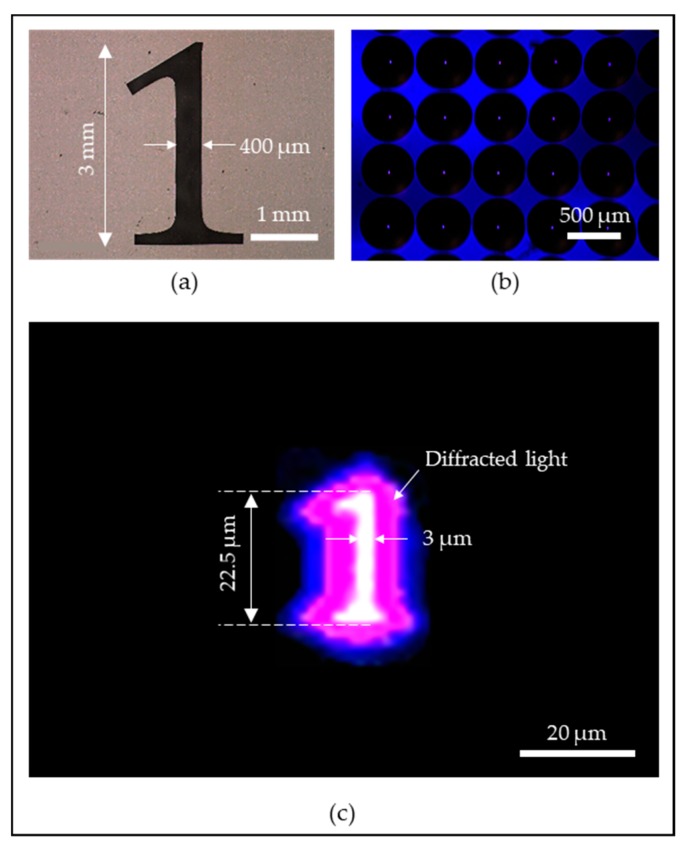
Real images generated by microlenses viewed by a microscope: (**a**) photomask pattern with number ‘1’; (**b**) mask pattern were imaged at the center of the MLAs; (**c**) close up view of the real image at the center of the microlens.

To demonstrate the functionality of the microlens array, a projection lithography scheme has been implemented where the main optical microlenses were fabricated by the TDTR process as described in Figure 8. The projection lithography is a type of photoreduction system in which a relatively large photopattern is projected into a smaller size pattern in microscale using a microlens. This technology is useful when an array of microscale patterns is needed. While the conventional system of the projection lithography used to have a single lens, the uniqueness of the system utilizes an array of the microlens, which produce the photo-reduced image in the form of an array. In this experiment, a photomask with pattern shape ‘1’ was placed above the UV light source to ensure the maximum amount of beam energy was passing through the mask pattern. A fabricated MLA with a diameter of 500 µm and a focal length of 450 µm was prepared using the TDTR process. The MLA was placed at a relatively long distance of 60 mm to the focal length, which was 133 times the focal length, so the incident light rays to the MLA can be considered close to the collimated state, which is desirable for the projection lithography process. An optical microscope was set above the MLA to monitor the photo-reduced images from the MLAs. The result from the projection lithography is presented in Figure 9. The photomask with pattern ‘1’ as shown in Figure 9a had a vertical line width of 400 µm and a length of 3 mm. Figure 9b shows the photo-reduced image through the microscope where an array of lenses and the projected images at the center of each microlens are shown. Figure 9c shows a close-up view from the one of the photo-reduced image formed by the microlens. The dimension of the brightest area of the projected image had a line width of 3 µm and a length of 22.5 µm, which matches with the theoretical reduction ratio of 133. 

**Figure 10:**
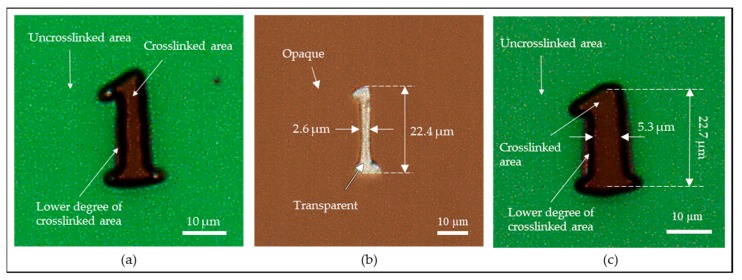
Results of the projection lithography experiment: (**a**) a photo-reduced pattern ‘1’ before etching process; (**b**) a photo-reduced pattern ‘1’ after etching process on a chromium glass; (**c**) an overexposed photo-reduced pattern ‘1’ with a measured linewidth of 5.3 µm before etching process.

The photo-reduced image was converted to a chromium glass to serve as a photomask with micropatterns to demonstrate the capability of the photomask fabrication. The chromium glass was spin-coated with positive photoresist, S1805, and loaded into the photoreduction system. The sample was UV-exposed for 15 s using 405 nm UVLED and followed by the development in the photoresist developer (Tetramethylammonium Hydroxide solution, MF 319, Rohm & Haas Electronics Materials LLC, Marlborough, MA, USA) for 1 min. The sample was rinsed with DI water and carefully dried with air to complete the process. Figure 10a shows the result of the photoreduction with pattern ‘1’ and Figure 10b shows the same photo-reduced pattern with the chromium etched out, which can be used as a photomask. The linewidth of the main body of the photo-reduced pattern ‘1’ was measured to be 2.6 µm, which had a 400 nm difference from the theoretically calculated linewidth as well as the measured linewidth as shown in Figure 9c. This gap can be explained by the resolution limitation of the wavelength of the UVLED (405 nm) that was used in the scheme. As the wavelength of the light source is limited to 405 nm, it is inevitable to have a 400 nm difference in the process. Regardless, this issue can be resolved by replacing the current 405 nm UVLED light source to another UV light source with a shorter wavelength. Another method is by reducing the intensity of the current light source and increase the exposure time, the drawback of this method is the increase of process time.

Another notable patterning difference occurred at the serifs located at the top and bottom of the photo-reduced pattern ‘1’ as shown in Figure 10b. The serifs are shortened because the linewidths are much smaller at the serifs, which resulted in weaker light intensity for crosslinking the photoresist. This phenomenon can also be noticed in the optical image as shown in Figure 9c. White, purple, blue, and black indicate the intensity of the light propagated through the pattern ‘1’ in a descending order, where white corresponds to the strongest light intensity and black corresponds to the weakest or no light intensity. The white area of the image as shown in Figure 9c well-matches with the crosslinked area as shown in Figure 10a. Similar relation can be applied to the other colors as well in Figure 9c where the purple area is corresponding to the lower degree of the crosslinked area as labeled in Figure 10a, and blue and black areas are corresponding to the uncrosslinked area. Note that the colors do not represent an exact value of the light intensity, it only provides an approximation about the strength of the light intensity, which can be used to estimate the result of the projection lithography experiment. This phenomenon explains the factor that caused the shortened serifs, which is still acceptable as the main body of the photo-reduced pattern ‘1’ is the main interest in this experiment. If the serifs were to be preserved, it would have resulted in a very undesirable result as shown in Figure 10c, where the linewidths of both the main body and the serifs of the photo-reduced pattern ‘1’ were enlarged due to overexposure. Therefore, in order to best preserve the details of the serifs (acute angle) while also obtain a desirable linewidth of 3 µm at the main body of pattern ‘1’, the experiment was conducted with low light intensity to maximally reduce the diffraction and sacrifice portion of the top and bottom serifs. 

Despite the differences mentioned, the proposed photoreduction system still demonstrated its capability in fabricating photomask with an array of photopatterns with a measured linewidth of 2.6 µm. To better understand how well the proposed system performed in comparison with the commercially available systems, recent researches were studied for comparison. A digital mirror device (DMD)-based projection lithography system was reported to have a resolution of 2.5 µm [23], and a cylindrical projection lithography system was reported to have a resolution of 5 µm [24]. Based on these two types of research’ results, the proposed projection lithography system demonstrated a 2.6 µm linewidth result, which is very competitive in terms of resolution, system cost, time of the process, and ease of use. This result shows great potential in photoreduction application for the MLA as the proposed system demonstrated a reduction ratio of 133 times from a 400 µm large pattern ‘1’ to a 2.6 µm photo-reduced pattern. This result can only be achieved as if the MLA was fabricated with high surface quality and uniformity, which indirectly shows the quality of the fabricated MLA using the presented TDTR process.

## 7. Conclusions

The timed-development-and-thermal-reflow (TDTR) process has demonstrated a novel fabrication process for optical microlens. The capability of the TDTR process presented different shapes of MLAs in terms of lens thickness, focal length, and lens aperture geometry with relatively simple process control of developing time and the microlens diameter. Different aperture geometries were introduced into microlens fabrication, which demonstrated the uniqueness of this fabrication process and also the flexibleness to fulfill different geometry limitations of the applied device. Nonlinear and linear regression analysis was introduced to model the profile of the microlens with developing time and the microlens diameter and converted into the optical parameters including lens thickness, lens diameter, focal length, and surface profile. The modeling equations were well aligned with experimental results in terms of the depth of meniscus trench and focal length, showing its reliability at predicting these values. The uniformity of the MLAs fabricated using this fabrication process was also discussed, up to 97.56% of uniformity was achieved which is very desirable when it comes to manufacturing. A projection lithography experiment was conducted, demonstrating a common application for MLAs. An array of pattern ‘1’ with a linewidth of 2.6 µm was successfully fabricated with only one single exposure. The result of the experiment demonstrated a reduction factor of 133 times for photoreduction application, wherein this particular case photomask fabrication. Having these qualities, this low-cost, fast-process, high uniformity and high throughput fabrication process with straight forward fabrication steps make it very compatible in various conditions. Furthermore, PDMS’s flexible and biocompatible characteristics and transparency to visible light and UV light make it very versatile and suitable for biomedical instruments, optoelectronics as well as small size electronics.

## Figures and Tables

**Table 1:** Summary of nonlinear regression analysis.

Coefficient	Estimate	Standard Error	*p*-Value
$b_{1}$	1.574077	0.07574	<0.0001
$b_{2}$	−1.089105	0.05139	<0.0001
$b_{3}$	1.078329	0.04032	<0.0001

**Table 2:** Summary of linear regression analysis.

Coefficient	Estimate	Standard Error	*p*-Value
$a_{0}$	−1.17852	0.16926	<0.0001
$a_{1}$	1.31930	0.08206	<0.0001
$a_{2}$	−0.27266	0.07436	<0.0001

**Table 3:** Estimated result of the constants using excel solver.

Coefficient	Estimate
*a*_0_	3.79583504
*a*_1_	0.742831171
*a*_2_	2.153050191
*a*_3_	2.407866931

**Table 4:** Uniformity of fabricated MLAs derived from measured lens thickness.

Aperture Geometry	Lens Thickness Mean	Variance	Standard Deviation	Uniformity (%)
Circular	139.25	24.58	4.96	96.44%
Square	137.07	11.20	3.35	97.56%
Hexagonal	98.38	65.88	8.12	91.75%
